# Ablation of Sphingosine 1-Phosphate Receptor Subtype 3 Impairs Hippocampal Neuron Excitability *In vitro* and Spatial Working Memory *In vivo*

**DOI:** 10.3389/fncel.2016.00258

**Published:** 2016-11-07

**Authors:** Daniela Weth-Malsch, Michiel Langeslag, Dimitra Beroukas, Luca Zangrandi, Iris Kastenberger, Serena Quarta, Philipp Malsch, Theodora Kalpachidou, Christoph Schwarzer, Richard L. Proia, Rainer V. Haberberger, Michaela Kress

**Affiliations:** ^1^Division of Physiology, Department of Physiology and Medical Physics, Medical University of InnsbruckInnsbruck, Austria; ^2^Anatomy and Histology and Centre for Neuroscience, Flinders UniversityAdelaide, SA, Australia; ^3^Department of Pharmacology, Medical University of InnsbruckInnsbruck, Austria; ^4^Genetics of Development and Disease Branch, National Institute of Diabetes and Digestive and Kidney DiseasesBethesda, MD, USA

**Keywords:** sphingosine 1-phosphate, S1P receptor 3, hippocampus, working memory, neuron excitability

## Abstract

Understanding the role of the bioactive lipid mediator sphingosine 1-phosphate (S1P) within the central nervous system has recently gained more and more attention, as it has been connected to major diseases such as multiple sclerosis and Alzheimer's disease. Even though much data about the functions of the five S1P receptors has been collected for other organ systems, we still lack a complete understanding for their specific roles, in particular within the brain. Therefore, it was the aim of this study to further elucidate the role of S1P receptor subtype 3 (S1P_3_) *in vivo* and *in vitro* with a special focus on the hippocampus. Using an S1P_3_ knock-out mouse model we applied a range of behavioral tests, performed expression studies, and whole cell patch clamp recordings in acute hippocampal slices. We were able to show that S1P_3_ deficient mice display a significant spatial working memory deficit within the T-maze test, but not in anxiety related tests. Furthermore, *S1p3* mRNA was expressed throughout the hippocampal formation. Principal neurons in area CA3 lacking S1P_3_ showed significantly increased interspike intervals and a significantly decreased input resistance. Upon stimulation with S1P CA3 principal neurons from both wildtype and ${\text{S1P}}_{3}^{-/-}$ mice displayed significantly increased evoked EPSC amplitudes and decay times, whereas rise times remained unchanged. These results suggest a specific involvement of S1P_3_ for the establishment of spatial working memory and neuronal excitability within the hippocampus.

## Introduction

The bioactive lysosphingolipid sphingosine 1-phosphate (S1P) plays an important role in the regulation of various cellular and physiological processes. Two sphingosine kinases (SphK1 and SphK2) phosphorylate sphingosine to form S1P, which can either act intracellularly or can be secreted to bind as an extracellular ligand to one of five G protein-coupled receptors (GPCR) of the S1P receptor family (S1P_1−5_) (reviewed in Strub et al., 2010; Maceyka et al., 2012). The different receptors activate G-proteins G_*i*_, G_*q*_, and G_12/13_ and their downstream signaling pathways. Deregulation of S1P metabolism and signaling occurs in various diseases as for example Alzheimer's disease (AD) and S1P receptors have been discovered as the target of several recently developed drugs (Couttas et al., 2014; van Echten-Deckert et al., 2014). One of these, FTY720, which originally has been launched for the treatment of multiple sclerosis, is currently also studied within the context of AD and has already been shown to attenuate beta-amyloid peptide (Aß42)-induced impairment of spatial learning and memory in rats (Asle-Rousta et al., 2013).

Our lab and others showed that S1P_1−3_ are expressed in primary afferent sensory neurons (Zhang et al., 2006; Kays et al., 2012). Of these S1P_1_ and S1P_3_ receptors play a significant role in regulating nociceptor function (Mair et al., 2011; Camprubí-Robles et al., 2013). In the central nervous system (CNS) S1P receptors are found with varying expression levels on different cell types and both astrocytes and neurons synthesize and release S1P (reviewed in (Soliven et al., 2011; Choi and Chun, 2013)). In neurons, S1P receptor signaling has been connected to proliferation, survival, and apoptosis, neurite extension, and retraction, excitability and neurotransmitter release (Okada et al., 2009). Furthermore, S1P signaling has been associated with learning and memory within the hippocampus (Kajimoto et al., 2007; Kanno et al., 2010; Akahoshi et al., 2011; Kanno and Nishizaki, 2011; Antonucci et al., 2012; Riganti et al., 2016).

Although there is growing evidence for the importance of S1P and its receptors for CNS processes, the specific functions of the individual receptors and their downstream signaling pathways are not yet fully understood. Therefore, it was the aim of this study to further elucidate the role of S1P_3_ in the CNS *in vivo* and *in vitro* using an S1P_3_ knock-out (${\text{S1P}}_{3}^{-/-}$) mouse model. We determined the expression of S1P_3_ within the hippocampal formation and compared memory- or anxiety-related behavioral phenotypes in wild-type (WT) and ${\text{S1P}}_{3}^{-/-}$ mice. Furthermore, using whole cell patch clamp recordings in acute hippocampal slice preparations we investigated excitability, evoked excitatory postsynaptic currents (eEPSCs) and ion channel expression in hippocampal principal neurons lacking S1P_3_ and compared these to WT control neurons.

## Materials and methods

### Ethics statement

All animal experiments were performed in accordance with national Austrian law and with permission of the Austrian Bundesministerium für Wissenschaft und Forschung (BMWF) (BMWF-66.011/0113-II/3b/2010). Every effort was taken to minimize the number of animals used in the experiments.

### Genetically modified mice

WT and ${\text{S1P}}_{3}^{-/-}$ mice were used for the experiments. Global S1P_3_ receptor null mutant mice (${\text{S1P}}_{3}^{-/-}$) have been described previously (Kono et al., 2004). Mice were fully inbred on a C57BL/6J background and each year the background strain was refreshed with C57BL/6J mice obtained from Charles River Laboratories. All behavioral experiments were performed in age-matched awake, unrestrained, male mice (12–16 weeks old), which were housed on a 12 h light/dark cycle with free access to food and water, unless stated otherwise. Experimenters were blinded to the genotype of the animal. All electrophysiological and qRT-PCR experiments were performed in mice of either sex (4–6 weeks old).

### qRT-PCR

Hippocampi were obtained as described previously (Spijker, 2011). In brief, mice were anesthetized using Isoflurane (Forane®) and decapitated using scissors. Brains were quickly removed and a fresh dissection of the hippocampus was performed. Therefore, the cerebellum was removed and the cortex was opened from the midline. The hippocampi were dissected and snap-frozen in liquid nitrogen and stored below −70°C for <1 month. Total RNA was extracted using peqGOLD TriFast (PeqLab, Germany). All samples were kept on dry ice until use and homogenized with plastic pestles in 1 ml TriFast. After 5 min incubation at RT, 200 μl of chloroform were added. Samples were briefly vortexed, incubated for 10 min at RT and centrifuged for 15 min at 4°C with 12,000 rcf. Fifteen milligram GlycoBlue Coprecipitant (Ambion) were added to the transferred aqueous phase before the addition of 500 μl of isopropanol. Samples were incubated for 10 min at RT and centrifuged for 10 min at 4°C with 12,000 rcf. The supernatant was mixed with 1 ml of 75% ethanol, briefly vortexed, and centrifuged for 5 min at 4°C with 12,000 rcf. After discarding the supernatant, the samples were centrifuged for 2 min under the same conditions. Residual ethanol was discarded and the pellet was air-dried before dissolving it in DEPC-treated MiliQ water. RNA quality and quantity was measured using a Nanodrop 2000 (PeqLab/Thermo Scientific).

For reverse transcription, 900 ng of total RNA were mixed with MuLv reverse transcriptase (2.5 U/μl; Applied Biosystems) with random hexamer primers (10 ng/μl), RiboLock (2 U/μl), 1x Taq Buffer (all from Thermo Scientific), MgCl_2_ (5 mM), and dNTPs (1 mM, both Fermentas) in a total volume of 20 μl. After 5 min incubation at RT, the mix was incubated for 15 min at 42°C, 5 min at 99°C, and 5 min at 5°C using a 2720 Thermal Cycler (Applied Biosystems).

For quantitative PCR, the RT-product was diluted 1:30 in autoclaved MilliQ-H_2_O. In total, 20 μl of reaction volume consisted of 9 μl diluted cDNA, 1x TaqMan^®;^ Gene Expression Assay and 1x TaqMan^®;^ Universal Master Mix II (no UNG, all Applied Biosystems). A list of all assays is provided in Supplementary Table 1. Reactions were performed in a MicroAmp Fast Optical 96-Well Reaction Plate (Applied Biosystems) using the 7500 Fast Real-Time PCR System (Applied Biosystems) for thermal cycling and real-time fluorescence measurements. The PCR cycle protocol consisted of 10 min at 95°C, 40 two-step cycles of 15 s each at 95°C and of 1 min at 60°C. No-template-controls and No-reverse-transcriptase-controls did not show amplification. Each sample was run in duplicates.

For the analysis of *S1p3*, threshold cycle (Cq) values were recorded as a measure of initial template concentration. Threshold was set to 0.2 of the baseline-corrected normalized reporter fluorescence (ΔRn) and baselines were automatically calculated by the 7500 software (Applied Biosystems). For samples that did not show amplification curves during the 40 PCR cycles Cq-values were artificially set to 40 for statistical analysis. Expression levels were calculated as following: ΔCq_*x, S1p3*_—geometrical mean (Cq_*x, Sdha*_, Cq_*x, Hprt*_, Cq_*x, Tfrc*_); ΔΔCq_*x*_ = ΔCq_*x*_—mean ΔCq of all WT samples; *S1p3* expression = 2^∧^-ΔΔCq_*x*_.

For the analysis of S1P receptors 1, 2, 4, and 5, and voltage-gated potassium channels, expression levels and primer efficiencies were analyzed using LinRegPCR (Version 2015.1) for hydrolysis probes [window of linearity: 4 points; parameters for calculation of mean efficiency: exclude no plateau samples, exclude efficiency outlier samples (>5% from group median), log-linear phase criterion: strictly continuous log-linear phase] (Ruijter et al., 2009; Tuomi et al., 2010). See Supplementary Table 1 for primer efficiencies. Expression levels (N0) were calculated by N0 = threshold/(Eff_meanĈq). Target expression levels were divided by the geometrical mean of *Sdha, Hprt* and *Tfrc* expression levels per sample. All target levels were then normalized to the mean target expression levels of the WT group. Outliers were identified using Grubbs' test (GraphPad Software, Inc.) and excluded from calculations.

### *In-situ* hybridization

The protocol was modified from an earlier method from Obernosterer et al. (2007) and Camprubí-Robles et al. (2013). Cryostat sections of murine hippocampi were Zamboni-fixed for 24 h, washed in phosphate buffered saline, digested with proteinase K (2 μg/ml), fixed with 4% PFA, acetylated (triethanolamine/HCl/acetic anhydride), and incubated with prehybridisation buffer (Roche). Target mRNAs were hybridized with 0.34 pmol of specific antisense double digoxigenin (DIG) labeled mRNA detection probes (S1P3, 5DigN/ACTGATGAGGAAGGCGATGTAT/3Dig_N) or scrambled probes (Exiqon, Copenhagen, Denmark) or hybridization buffer as negative controls overnight at 55°C (Camprubí-Robles et al., 2013). Washing with SSC was followed by blocking and incubation with alkaline phosphatase coupled anti-DIG antiserum. Washing in PBS followed incubation with secondary antisera for 1 h. Slides were incubated with BCIP/NBT (Roche) to visualize *S1p3* receptor mRNA, washed in PBS and coverslipped in buffered glycerol. Images were taken using a brightfield microscope (BX50, Olympus).

### Behavioral testing

#### T-maze

Mice were tested in the T-maze rewarded alternation paradigm in an enclosed maze as described in the protocol by Deacon and Rawlins (2006). Briefly, during a habituation period of 5 days mice were accustomed to the touch and handling by the experimenter and to the food reward in their home cage. In a second step food was rationed so that mice were kept at 90–95% of their free-feeding body weight, which was controlled by daily measurement. On the last 2 days of the habituation period, mice were habituated to the T-maze by letting them explore the maze and find the food rewards first without any arm being closed and then with one arm being closed. This was performed four times for each mouse, with intervals of 10 min. On testing days, animals were transferred into the testing room 5–10 min before testing in order to achieve an optimal state of arousal. On five consecutive days each mouse was subjected to 10 trial runs. For each trial run sample and choice arm were chosen randomly and both arms were baited with a reward. Then a door blocked the choice arm and the mouse was transferred into the start area in order to allow them to explore the sample arm and consume the reward. Immediately afterwards the mouse was placed into the start area again, facing away from the arms, and the door of the choice arm was removed so that the mouse could enter both arms. If the mouse directly entered into the choice arm the trial was recorded as correct choice, if it entered the sample arm again the trial was counted as wrong choice. Mice were also tested in the spontaneous alternation paradigm. For this paradigm no habituation was performed, as it is based on the novelty of the maze and the explorative drive of rodents. For each mouse one trial was performed. The mouse was placed into the start area with both arms open and was allowed to choose a goal arm. This arm was gently closed behind the mouse. After 30 s the mouse was removed, the goal arm was opened again and the mouse was placed into the start area facing away from the arms. Again it was allowed to choose between the two open arms.

#### Weight analysis

For the analysis of body weight, mice were weighed weekly starting after weaning until they reached an age of 30 weeks.

#### Home cage activity

Mice were monitored in their home cages using the Infra-Mot system (TSE, Bad Homburg, Germany) for three dark and two light phases to record their basal activity and circadian rhythm.

#### Barnes maze

A white circular arena (96 cm diameter) without walls with 20 circular holes (3.5 cm diameter) that were equally distributed around the perimeter was set up at a height of 120 cm. The escape box made out of black plastic could be positioned under any of the holes. The remaining holes could be closed by small lids. The maze was placed in the center of the testing room, illuminated to 60 lx and four spatial cues were positioned around the maze. Mice were transferred into the anteroom of the testing room 24 h before habituation to the maze. For each mouse included in the experiment, a different target hole was chosen and remained the same throughout the entire experiment. During habituation each mouse was placed onto the maze for 5 min to explore the maze and find both the target hole and escape box for the first time. In case it did not find the target hole, the experimenter gently guided it there. After that, the mouse was habituated to the escape box for 2 min. The habituation day was followed by 4 days of acquisition (day 1–day 4), each consisting of 3 trials. During the trials, each mouse was placed onto the maze for 3 min and given the chance to explore the maze and find the target hole with the escape box. The trials were monitored using the Video-Mot 2 equipment and software (TSE-systems, Bad Homburg, Germany). The number of primary errors (visits to wrong holes before finding the target hole), the primary latency (time until reaching the target hole) and the primary path length (path traveled until reaching the target hole) were analyzed. On days 5 and 12 probe trials were performed to test short and long-term memory. The target hole was closed, the mouse was placed onto the maze for 5 min and later the maze was divided into four quadrants and the time spent in each quadrant was analyzed. The target hole was always in the center of quadrant 1.

#### Open field, elevated plus maze, and light-dark test

The open field, elevated plus maze and light-dark tests were performed as previously published (Wittmann et al., 2008) and in accordance with the recommendations of EMPRESS (European Mouse Phenotyping Resource of Standardized Screens; http://empress.har.mrc.ac.uk). Twenty four hour before the beginning of experiments mice were transferred to the anteroom of the testing room. Free access to food and water and the light-dark cycle were maintained. All tests were video monitored using the Video-Mot 2 equipment and software (TSE-systems, Bad Homburg, Germany). Mice were subjected to the tests in the following order: open field, elevated plus maze and light-dark test. Between the tests there were 3 test free days each. Open field arenas consisted of a size of 50 × 50 cm, were illuminated to 150 lx and subdivided into three zones: border zone (8 cm along the walls), intermediate zone and central zone (central 16% of overall arena). Exploratory activity was observed for 10 min. The elevated plus maze with two enclosed arms (20 cm walls) and two open arms, each 50 × 5 cm in size, was elevated about 80 cm above ground. Exploratory activity was monitored in the open arms for 5 min at 180 lx. Finally, the light-dark test was performed in the same arenas as the open field with a black box covering 1/3 of the area and exploratory activity was monitored for 10 min at 400 lx.

### Electrophysiology

#### Preparation of acute hippocampal slices

Transversal hippocampal slices were prepared as described previously (Bischofberger et al., 2006). Mice of a minimum age of 4 weeks were chosen to ensure completed hippocampal development. Mice were anesthetized with Isoflurane (Forane®) and decapitated using scissors. Brains were quickly removed and placed into ice cold sucrose-based cutting/storage solution containing (in mM): sucrose 75, glucose 10, NaCl 87, NaHCO_3_ 25, NaH_2_PO_4_ 1.25, KCl 2.5, MgCl_2_ 7, and CaCl_2_ 0.5, equilibrated with 95% O_2_ and 5% CO2, osmolarity: ~320 mOsm, pH 7.4. The two hemispheres were cut at the midsagittal line, placed with the sagittal plane facing down and a cut tangential to the dorsal surface and perpendicular to the sagittal plane, was used to cut away the dorsal part of the brain to conserve the integrity of the mossy fiber tract (Bischofberger et al., 2006). Transversal slices (300 μm thick) were prepared using a vibratome (VT 1200S, Leica Microsystems). Slices were kept at ~34°C for 30 min and thereafter at room temperature (RT) in sucrose-based cutting/storage solution.

#### Patch-clamp recordings

Slices were transferred to a recording chamber mounted on a Zeiss Axioskop 2 FS mot and continuously perfused with artificial cerebrospinal fluid (aCSF) containing (in mM): glucose 25, NaCl 125, NaHCO_3_ 25, NaH_2_PO_4_ 1.25, KCl 2.50, MgCl_2_ 1, CaCl_2_ 2, equilibrated with 95% O_2_ and 5% CO_2_, osmolarity: ~320 mOsm, pH 7.4, flow rate: 2–3 ml/min. Patch pipettes were filled with intracellular solution containing (in mM): K^+^-gluconate 135, KCl 20, EGTA 0.1, MgCl_2_ 2, Hepes 10, NaGTP 0.3, MgATP 2, osmolarity: ~295 mOsm, pH 7.3. Pipette resistance was 4–5 MΩ after filling. For data aquisition EPC10 amplifier and software (PATCHMASTER, HEKA Elektronik, Lambrecht/Pfalz, Germany) were used. Experiments were performed at room temperature and the amplifier software automatically corrected the junction potential, which was 23.72 ± 0.52 mV. Data were filtered using a Bessel filter at 2.9 kHz and the sampling rate ranged from 4 to 50 kHz depending on the protocol applied. CA3 pyramidal neurons were maintained at −70 mV holding potential in the whole cell voltage clamp configuration of the patch-clamp technique and at 0 pA in current clamp configuration. At the beginning of every recording, passive membrane properties (series resistance, capacitance and input resistance) were determined using a block pulse protocol of +10 mV and 30 ms. Series resistance was between 12 and 30 MΩ and not routinely compensated. For recordings in the voltage clamp configuration, series resistance was monitored regularly throughout the experiment. For current clamp recordings, series resistance was monitored at the beginning and at the end of the experiment. Duration of the recordings was up to 45 min and series resistance change over time usually was about 10%. The resting membrane potential (RMP) was determined in current clamp mode, and recordings were discarded if RMP was above −50 mV or if cells displayed spontaneous action potential firing.

#### Assessment of basic electrophysiological properties

In order to determine rheobase and action potential (AP) parameters depolarizing current steps (30 pA increase, 500 ms duration) starting at −240 pA were applied. AP analysis was performed as follows and as it has been described previously (Sørensen et al., 2011; Langeslag et al., 2014). The first three evoked action potentials were averaged. To achieve this, trace fit functions within the FITMASTER software (HEKA Elektronic, Lambrecht/Pfalz, Germany) and Microsoft Excel for further mathematical calculations were used. AP overshoot, afterhyperpolarization (AHP) and the AHP time constant (τ_AHP_) were measured. AP threshold and the speed of both depolarization and repolarization were derived from the 1st derivative of the AP. For AP duration, the half-amplitude was calculated and duration at this value was measured. Two ramp-shaped current stimuli (5 s duration) based on 1x rheobase and 2x rheobase were applied to analyze the number of action potentials and the duration of interspike intervals. For current—frequency analysis frequency was defined as 1/interspike interval and plotted against the relative current intensity from 0 to 200% of rheobase. In order to test the influence of S1P, RMP was recorded for 10 min under control conditions (aCSF) followed by 10 min application of S1P (1 μM). Following this chemical stimulus, the step- and the ramp-shaped current stimulation protocols were repeated.

#### Evoked excitatory postsynaptic currents (eEPSCs)

For the recording of eEPSCs a bipolar tungsten stimulation electrode was placed in the granule cell layer/hilus and constant-current pulses (width 0.1 ms, 0.05 Hz) were applied. To pharmacologically isolate non-NMDA receptor mediated EPSCs the GABA_*A*_ receptor antagonist picrotoxin (100 μM) and the NMDA receptor antagonist D-APV (100 μM) were added to the aCSF. eEPSCs were recorded in voltage clamp mode for 10 min, followed by 10 min with additional application of S1P (1 μM) and another 10 min under control conditions. eEPSC amplitudes were normalized to the mean eEPSC amplitude during the first 10 min of the recording. For the analysis of 10–90% rise and decay times, the rise time function within the trace fit functions of FITMASTER was used.

### Statistical analysis

For detailed statistical analysis GraphPad Prism 5 (GraphPad Software, Inc.) was used. Data are presented as mean ± SEM. Box plots extend from 25 to 75th percentile and display median (line), mean (cross) and whiskers (Tukey method). Scatter dot plots present individual data points and median with interquartile range. Two-way repeated measures ANOVA followed by Bonferroni *post hoc* tests, two-way ANOVA followed by Bonferroni *post hoc* test and Mann-Whitney *U*-test for comparisons between groups were used. Differences were considered statistically significant at *p* < 0.05.

## Results

**Figure 1:**
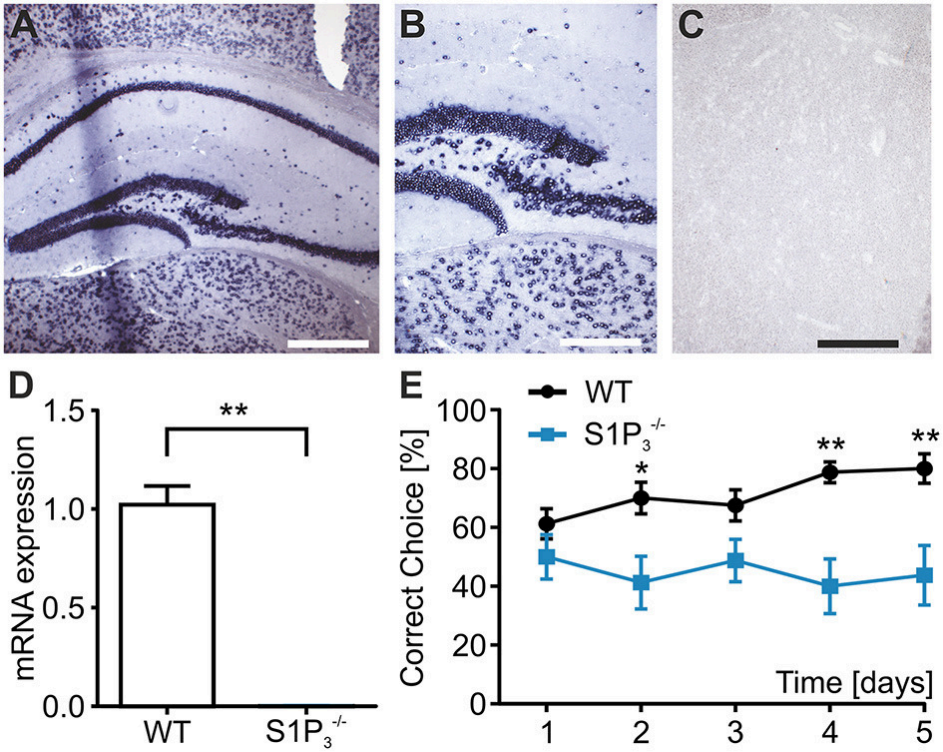
**${\text{S1P}}_{3}^{-/-}$ mice displayed working memory deficit and no expression of ***S1p3*** mRNA in hippocampal principal cells. (A–C)** *In situ* hybridization of mouse hippocampus. **(A)** *In situ* hybridization for the S1P_3_ receptor subtype in mouse hippocampus showed strong staining of the areas CA1–CA3 as well as the dentate gyrus. Bar = 400 mm **(B)** Higher magnification shows intense perinuclear staining at the border between CA3 and dentate gyrus. Bar = 200 mm **(C)** Incubation with a DIG-labeled scrambled probe gave no signal. Bar = 200 mm. **(D)** *S1p3* mRNA was expressed in hippocampi from WT mice while ${\text{S1P}}_{3}^{-/-}$ mice showed no hippocampal expression of *S1p3* mRNA (Mann-Whitney *U*-test: ^**^p < 0.01, *n* = 6). **(E)** ${\text{S1P}}_{3}^{-/-}$ mice showed significant impairment in the T-maze rewarded alternation spatial working memory task when compared to WT control mice (two-way repeated measures ANOVA with Bonferroni post-tests: ^*^p < 0.05, ^**^p < 0.01, *n* = 8).

S1P_3_ is expressed in the central nervous system both in rodents and humans (Yamaguchi et al., 1996; Zhang et al., 1999) including the hippocampus, whose function is required for task learning in the rewarded alternation T-maze test (Deacon et al., 2002; Deacon and Rawlins, 2006). Therefore, we investigated the expression of S1P_3_ in hippocampi of WT and ${\text{S1P}}_{3}^{-/-}$ mice. To assess location and expression pattern of *S1p3*-mRNA in mouse hippocampus, *in-situ* hybridization was used since indirect immunofluorescence did not provide unequivocal results due to the lack of selective antibodies. Expression of *S1p3* mRNA was present predominantly in the pyramidal cell layer and the staining pattern supported expression in neurons throughout the hippocampal formation (Figures 1A–C). qRT-PCR measurements, clearly demonstrated the expression of *S1p3* mRNA in hippocampi from WT mice, whereas no expression of *S1p3* mRNA was detectable in hippocampi from ${\text{S1P}}_{3}^{-/-}$ mice (Figure 1D). No significant compensatory change in expression levels were detected of the other four S1P receptors in ${\text{S1P}}_{3}^{-/-}$ mice (Table 1).

**Table 1:** **Expression levels of S1P receptors 1, 2, 4, and 5 in hippocampi from WT and ${\text{S1P}}_{3}^{-/-}$ mice**.

**Gene name**	**WT**	**${\text{S1P}}_{3}^{-/-}$**	***p*-value**
*S1pr1*	1 ± 0.037404	1.097586 ± 0.178521	0.6286
*S1pr2*	1 ± 0.091626	1.057782 ± 0.094352	0.8286
*S1pr4*	1 ± 0.0066300	1.353522 ± 0.206668	0.4857
*S1pr5*	1 ± 0.150916	1.367538 ± 0.114230	0.2000

Data are presented as mean ± SEM (Mann-Whitney U-test; n = 4)

### Function of S1P receptor 3 in formation of working memory

We applied a panel of behavioral experiments including basic observations of weight development, circadian rhythm, memory related tests (T-maze, Barnes maze) and several anxiety related tests (open field, elevated plus maze, light-dark test). As previously published by other groups, mice lacking the S1P_3_ receptor did not show any obvious phenotypic abnormalities when compared to WT littermates, that is they were indistinguishable from their WT littermates and displayed a comparable overall health, fertility and longevity (Ishii, 2001; Kono et al., 2004).

In order to specifically assess the function of S1P_3_ in memory formation and retrieval, we performed a T-maze test using the rewarded alternation paradigm, a simple and sensitive test based on an appetitive motivation in rodents to discover cognitive dysfunction (Deacon and Rawlins, 2006). During a course of 5 trial days WT mice displayed normal task learning, whereas ${\text{S1P}}_{3}^{-/-}$ mice displayed a significant learning impairment (correct choice: trial day 1: WT: 61.3 ± 5.2%, ${\text{S1P}}_{3}^{-/-}$: 50.0 ± 7.6%; trial day 5: WT: 80.0 ± 5.0%, ${\text{S1P}}_{3}^{-/-}$: 43.8 ± 10.2%; *p* < 0.05; two-way repeated measures ANOVA, Figure 1E). Using the spontaneous alternation paradigm, which tests whether the novelty of the maze leads to spontaneous exploration and alternation of choice arms or whether mice display a side preference, ${\text{S1P}}_{3}^{-/-}$ and WT mice demonstrated comparable normal choice alternation rates of 75% (*n* = 8).

**Figure 2:**
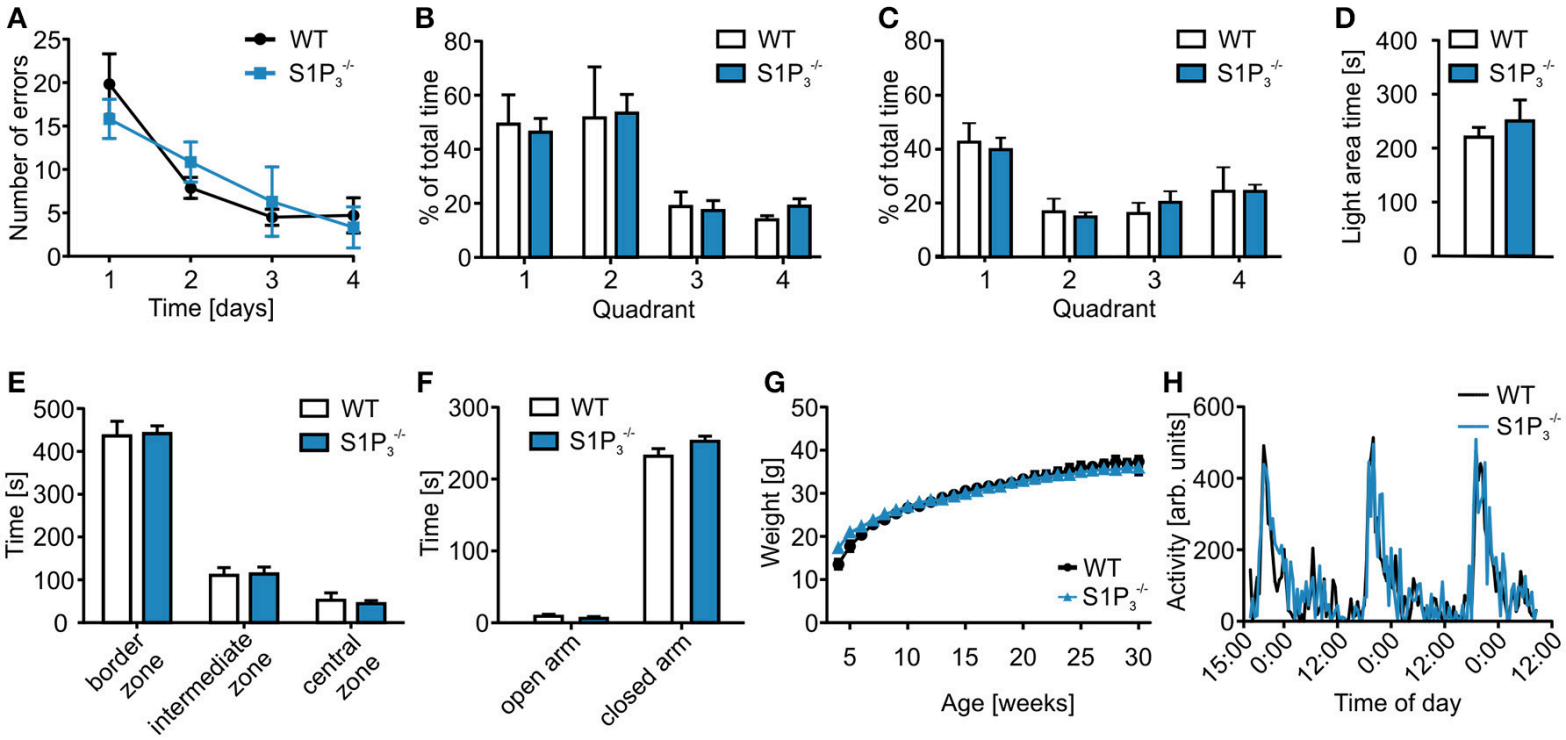
**${\text{S1P}}_{3}^{-/-}$ mice displayed no deficits in further behavioral assessment. (A–C)** Barnes maze test of ${\text{S1P}}_{3}^{-/-}$ and WT mice. **(A)** ${\text{S1P}}_{3}^{-/-}$ mice neither differed significantly from WT mice in the Barnes maze spatial reference memory task during the training period (two-way repeated measures ANOVA: n.s., WT: *n* = 6, ${\text{S1P}}_{3}^{-/-}$: *n* = 10) nor in the **(B)** short-term memory test on day 5 (two-way repeated measures ANOVA: n.s., WT: *n* = 6, ${\text{S1P}}_{3}^{-/-}$: *n* = 10) or in the **(C)** long-term memory test on day 12 (two-way repeated measures ANOVA: n.s., WT: *n* = 6, ${\text{S1P}}_{3}^{-/-}$: *n* = 10). **(D)** ${\text{S1P}}_{3}^{-/-}$ mice spent a similar amount of time in the open area during the light-dark test (Mann-Whitney *U*-test: n.s., WT: *n* = 8, ${\text{S1P}}_{3}^{-/-}$: *n* = 9). **(E)** ${\text{S1P}}_{3}^{-/-}$ mice spent similar amounts of time in the different zones of the open field test as WT control mice (two-way ANOVA: n.s., WT: *n* = 8, ${\text{S1P}}_{3}^{-/-}$: *n* = 9). **(F)** ${\text{S1P}}_{3}^{-/-}$ mice spent similar amounts of time on the open and closed arms of the elevated plus maze (two-way ANOVA: n.s., WT: *n* = 8, ${\text{S1P}}_{3}^{-/-}$: *n* = 9). **(G)** ${\text{S1P}}_{3}^{-/-}$ and WT littermates showed similar weight gain over a course of 30 weeks after birth (two-way repeated measures ANOVA: n.s., WT: *n* = 5, ${\text{S1P}}_{3}^{-/-}$: *n* = 10). **(H)** ${\text{S1P}}_{3}^{-/-}$ and WT mice displayed similar circadian rhythm in a home cage activity test (two-way repeated measures ANOVA: n.s., *n* = 4).

In contrast to their performance deficit in the T-maze test, ${\text{S1P}}_{3}^{-/-}$ mice showed no significant differences in the spatial reference memory Barnes maze test, neither during the acquisition phase on days 1–4 nor during the probe trials on days 5 and 12 (primary errors: day 1: WT: 19. 83 ± 3.49, S1P${\text{S1P}}_{3}^{-/-}$: 15.83 ± 2.25; day 4: WT: 4.72 ± 2.01, ${\text{S1P}}_{3}^{-/-}$: 3.33 ± 2.37; two-way repeated measures ANOVA: n.s.; time spent in quadrant 1: day 5: WT: 49.29 ± 10.85%, ${\text{S1P}}_{3}^{-/-}$: 46.29 ± 5.16%; day 12: WT: 42.62 ± 7.04%, ${\text{S1P}}_{3}^{-/-}$: 39.84 ± 4.37%; two-way repeated measures ANOVA: n.s.; Figures 2A–C). Furthermore, no differences could be seen when comparing the primary latency and the primary path length during the acquisition phase (primary latency: day 1: WT: 145.06 ± 16.96 s, ${\text{S1P}}_{3}^{-/-}$: 152.03 ± 10.11 s; day 4: WT: 16.36 ± 2.99 s, ${\text{S1P}}_{3}^{-/-}$: 22.33 ± 11.00 s; two-way repeated measures ANOVA: n.s.; primary path length: day 1: WT: 779.97 ± 94.56 cm, ${\text{S1P}}_{3}^{-/-}$: 762.97 ± 95.22 cm; day 4: WT: 150.30 ± 34.28 cm, ${\text{S1P}}_{3}^{-/-}$: 151.58 ± 64.52 cm; two-way repeated measures ANOVA: n.s.).

In a series of anxiety related tests, no significant differences were observed (Figures 2D–F). In the light-dark test ${\text{S1P}}_{3}^{-/-}$ mice spent similar amounts of time in the light compartment (light area time: WT: 220.5 ± 17.80 s, ${\text{S1P}}_{3}^{-/-}$: 250.6 ± 38.76 s; Mann-Whitney *U*-test: n.s.; Figure 2D) as well as in the three different zones in the open field test (border zone: WT: 436.65 ± 34.05 s, ${\text{S1P}}_{3}^{-/-}$: 441.50 ± 18.43 s; intermediate zone: WT: 110.77 ± 17.64 s, ${\text{S1P}}_{3}^{-/-}$: 114.14 ± 16.03 s; central zone: WT: 52.53 ± 17.24 s, ${\text{S1P}}_{3}^{-/-}$: 44.31 ± 7.04 s; two-way repeated measures ANOVA: n.s.; Figure 2E). Furthermore, ${\text{S1P}}_{3}^{-/-}$ mice showed normal behavior in the elevated plus maze (open arm: WT: 9.19 ± 2.68 s, ${\text{S1P}}_{3}^{-/-}$: 6.13 ± 2.55 s; closed arm: WT: 231.82 ± 10.67 s, ${\text{S1P}}_{3}^{-/-}$: 252.74 ± 7.03 s; two-way ANOVA: n.s.; Figure 2F).

${\text{S1P}}_{3}^{-/-}$, ${\text{S1P}}_{3}^{+/-}$ and WT littermates also displayed similar weight development (week 4: WT: 13.57 ± 1.11 g, ${\text{S1P}}_{3}^{+/-}$: 15.05 ± 0.72 g, ${\text{S1P}}_{3}^{-/-}$: 17.28 ± 0.59 g; week 30: WT: 37.38 ± 1.18 g, ${\text{S1P}}_{3}^{+/-}$: 35.66 ± 1.33 g, ${\text{S1P}}_{3}^{-/-}$: 35.89 ± 0.97 g; two-way repeated measures ANOVA: n.s.; Figure 2G), also circadian rhythms did not differ between ${\text{S1P}}_{3}^{-/-}$ and WT mice (two-way repeated measures ANOVA: n.s.; Figure 2H).

Together these results are suggesting a specific role of S1P_3_ receptor for appetitively motivated memory formation in the hippocampus.

### Alteration of general electrophysiological properties in S1P receptor 3 depleted neurons

**Figure 3:**
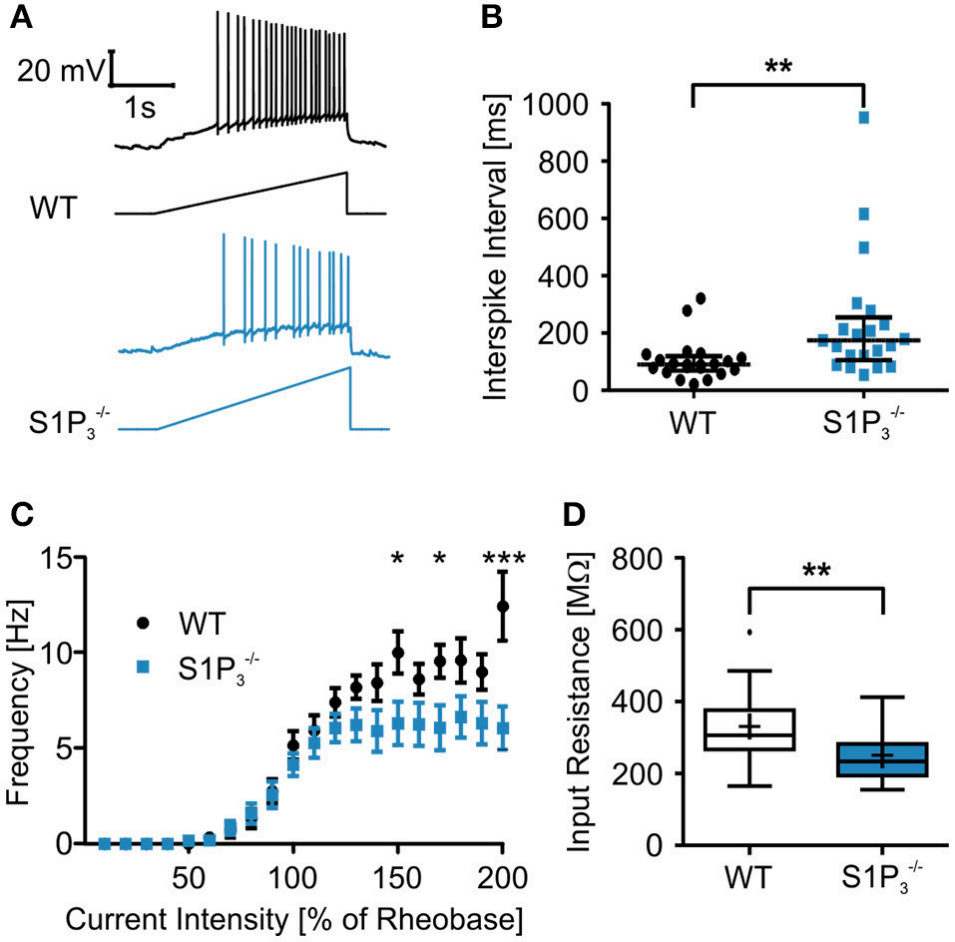
**Principal neurons in hippocampal area CA3 of ${\text{S1P}}_{3}^{-/-}$ mice displayed higher interspike intervals and a lower input resistance the WT control neurons. (A)** Representative traces of whole cell current clamp recordings of principal neurons in area CA3 of acute hippocampal slices from WT and ${\text{S1P}}_{3}^{-/-}$ mice during ramp based on 2x rheobase. ${\text{S1P}}_{3}^{-/-}$ neurons showed significant higher interspike intervals. **(B)** Quantification of recordings represented in **(A)** (Mann-Whitney *U*-test: ^**^p < 0.01, *n* = 21). **(C)** Current—frequency (I-f) relationship. Frequency increases with current intensity with a significantly stronger increase in WT neurons. (two-way ANOVA: interaction *p* = 0.0002, genotype *p* < 0.0001, current intensity *p* < 0.0001, Bonferroni post-tests: ^*^p < 0.05, ^***^p < 0.001, *n* = 21) **(D)** Principal neurons in area CA3 of acute hippocampal slices from ${\text{S1P}}_{3}^{-/-}$ mice had a significant lower input resistance than WT control neurons (Mann-Whitney *U*-test: ^**^p < 0.01, *n* = 21).

Since we observed significant differences between the two genotypes within the hippocampal based T-maze test, we explored basic electrophysiological properties of hippocampal principal neurons *in situ* using whole cell patch clamp recordings on acute hippocampal slices. We concentrated on hippocampal area CA3 as it was demonstrated previously that S1P_3_ translocates to presynaptic nerve terminals upon stimulation with S1P in acutely dissociated CA3 pyramidal neurons (Kanno et al., 2010), suggesting a specific role of this receptor in CA3 but not in CA1 neurons. The following parameters were investigated: series resistance, capacitance, input resistance, rheobase, AP threshold, AP overshoot, AP afterhyperpolarization, τ_AHP_, AP amplitude, AP half duration, AP depolarization and repolarization speed, AP frequency and AP interspike interval (see Table 2). Neurons with a deficiency of S1P_3_ exhibited significantly longer and less regular interspike intervals as compared to WT control neurons (WT: 105.3 ± 15.7 ms, ${\text{S1P}}_{3}^{-/-}$: 235.1 ± 46.7 ms; Mann-Whitney *U*-test: *p* = 0.0021; Figures 3A,B). No differences within the other AP parameters were detected (Table 2). When we compared the current—firing frequency relationships, we observed that in both genotypes firing frequency increases with increasing current intensity, but the increase was significantly stronger in WT neurons (at current intensity 200% of rheobase: WT: 12.44 ± 1.802 Hz, ${\text{S1P}}_{3}^{-/-}$: 6.062 ± 1.132 Hz; two-way ANOVA: interaction: *p* = 0.0002, genotype: *p* < 0.0001, current intensity *p* < 0.0001, Bonferroni post-test: *p* < 0.001; Figure 3C). Furthermore, ${\text{S1P}}_{3}^{-/-}$ neurons displayed a significantly decreased input resistance (WT: 331.2 ± 21.6 MΩ, ${\text{S1P}}_{3}^{-/-}$: 251.3 ± 16.8 MΩ; Mann-Whitney *U*-test: *p* = 0.0048; Figure 3D). Taken together these facts hinted toward a decreased neuronal excitability, which could be due to a change in potassium conductance and a possible deregulation of potassium channel expression.

**Table 2:** **Basic electrophysiological properties of principal neurons in area CA3 in acute hippocampal slices from WT and ${\text{S1P}}_{3}^{-/-}$ mice**.

**CA3**	**WT (*n* = 21)**	**${\text{S1P}}_{3}^{-/-}$ (*n* = 21)**	***p*-value**
Series resistance [MΩ]	19.6±0.8	21.6±0.8	0.0994
Capacitance [pF]	137.9±6.5	123.2±62	0.0741
Input resistance [MΩ]	331.2±21.6	251.3±16.8	0.0048^**^
Resting membrane potential [mV]	−66.1±1.4	−65.2±1.5	0.6689
Rheobase [pA]	125.7±11.1	150.0±19.5	0.4256
AP no. in ramp based on 1x rheobase	10.2±1.5	7.5±1.0	0.2714
Interspike intervals [ms]	167.2±14.1	268.1±58.8	0.0536
AP no. in ramp based on 2x rheobase	23.8±1.6	18.8±2.3	0.0659
Interspike intervals [ms]	105.3±15.7	235.1±46.7	0.0021^**^
AP threshold [mV]	−37.3±1.0	−39.2±1.0	0.2085
AP overshoot [mV]	57.7±0.6	56.8±1.2	0.9298
AP afterhyperpolarization [mV]	−50.0±0.7	−50.7±0.6	0.2794
τ_*AHP*_ [ms]	3.1±0.48	3.3±0.36	0.3024
AP amplitude [mV]	94.9±1.5	96.0±1.8	0.5294
AP duration [ms]	1.1±0.08	1.1±0.06	0.2905
Depolarization speed [mV/ms]	449.4±26.5	414.3±25.5	0.3651
Repolarization speed [mV/ms]	−74.3±3.9	−70.6±3.2	0.4812

Data are presented as mean ± SEM (Mann-Whitney U-test:

** *p <0.01). No correction for multiple comparisons has been performed. Bold values are statistically significant values*.

Based on a literature research on potassium channels associated with S1P signaling (Takabe et al., 2008; Benamer et al., 2009, 2011) and hippocampal expression of potassium channel α subunits as published in the Allen Mouse Brain Atlas [(Lein et al., 2006), Website: © 2015 Allen Institute for Brain Science. Allen Mouse Brain Atlas [Internet]. Available from: http://mouse.brain-map.org], we selected nine potassium channel candidates (*Kcnj3, Kcnj6, Kcnj8, Kcnj9, Kcna4, Kcnc3, Kcnc4, Kcnd2, Kcnd3*) for mRNA quantification in hippocampi of WT and ${\text{S1P}}_{3}^{-/-}$ mice. None of the potassium channels tested displayed a significant deregulation in ${\text{S1P}}_{3}^{-/-}$ mice (Table 3).

**Table 3:** **Potassium channel mRNA expression levels in hippocampi from WT and ${\text{S1P}}_{3}^{-/-}$ mice**.

**Gene name**	**WT**	**${\text{S1P}}_{3}^{-/-}$**	***p*-value**
*Kcnj3*	1 ± 0.076595	1.101751 ± 0.031515	0.5281
*Kcnj6*	1 ± 0.104304	1.115369 ± 0.111236	0.5714
*Kcnj8*	1 ± 0.041285	0.980659 ± 0.09296	0.5714
*Kcnj9*	1 ± 0.070697	1.073008 ± 0.074166	0.3867
*Kcna4*	1 ± 0.102338	0.919057 ± 0.155438	>0.9999
*Kcnc3*	1 ± 0.125807	1.192529 ± 0.102352	0.2389
*Kcnc4*	1 ± 0.072289	1.063664 ± 0.055906	0.4285
*Kcnd2*	1 ± 0.109368	1.097379 ± 0.089368	0.6753
*Kcnd3*	1 ± 0.134945	0.985784 ± 0.119652	>0.9999

*Data are presented as mean ± SEM (Mann-Whitney U-test; n = 6–10)*.

### S1P modulated electrophysiological properties of principal neurons in hippocampal area CA3

**Figure 4:**
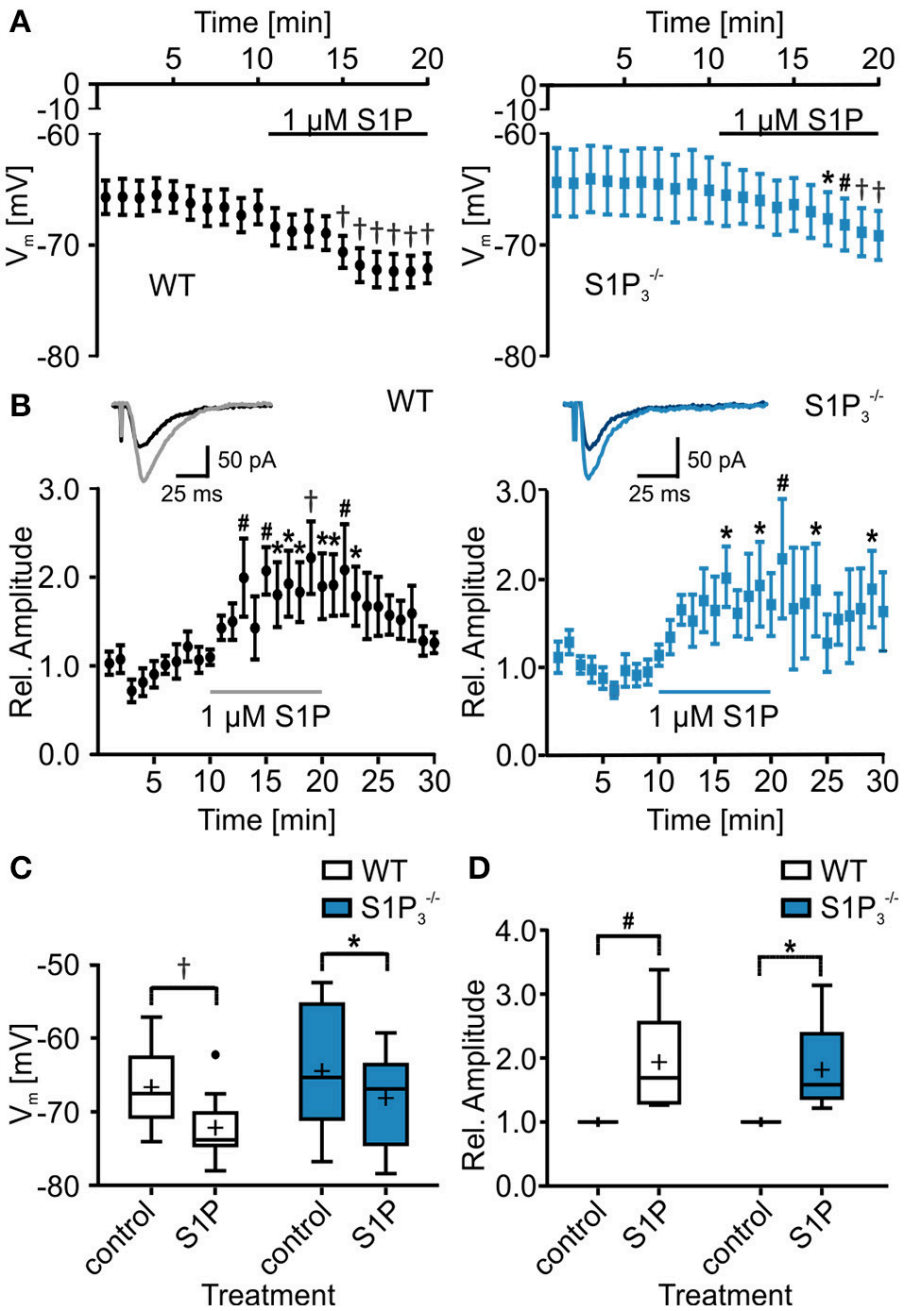
**Application of S1P led to decrease in resting membrane potential and increase in eEPSC amplitude. (A)** Whole cell current clamp recordings of principal neurons in area CA3 of acute hippocampal slices from WT and ${\text{S1P}}_{3}^{-/-}$ mice. Both genotypes reacted to application of S1P with a significant reduction in resting membrane potential, with a slightly stronger effect in WT neurons (two-way repeated measures ANOVA with Bonferroni post-tests compared to control mean: ^*^p < 0.05, ^#^*p* < 0.01, ^†^*p* < 0.001, WT: *n* = 10, ${\text{S1P}}_{3}^{-/-}$: *n* = 8). **(B)** Whole cell voltage clamp recordings of principal neurons in area CA3 with mossy fiber stimulation. Inserts show representative traces of eEPSCs recorded before and during S1P application. Application of S1P led to significant increase in eEPSC amplitude in both genotypes with a slightly stronger effect in WT neurons (two-way repeated measures ANOVA with Bonferroni post-tests compared to control mean: ^*^p < 0.05, ^#^*p* < 0.01, ^†^*p* < 0.001, WT: *n* = 6, ${\text{S1P}}_{3}^{-/-}$: *n* = 5). **(C)** Quantification of **(A)** (two-way repeated measures ANOVA with Bonferroni post-tests: ^*^p < 0.05, ^†^*p* < 0.001). **(D)** Quantification of **(B)** (two-way repeated measures ANOVA with Bonferroni post-tests: ^*^p < 0.05, ^#^*p* < 0.01).

Since S1P is the main ligand for the S1P receptors, we also tested the effect of S1P application on basic electrophysiological properties in CA3 principal neurons. Both in WT neurons and neurons lacking S1P_3_ the resting membrane potential hyperpolarized, with a slightly weaker effect in ${\text{S1P}}_{3}^{-/-}$ neurons (RMP: WT control: −66.69 ± 1.66 mV, WT with S1P: −72.17 ± 1.43 mV, ${\text{S1P}}_{3}^{-/-}$ control: −6450 ± 3.01 mV, ${\text{S1P}}_{3}^{-/-}$ with S1P: −68.17 ± 2.27 mV; two-way repeated measures ANOVA: treatment *p* < 0.0001, genotype *p* = 0.2904, interaction *p* = 0.2694, Bonferroni post-tests: WT *p* < 0.001, ${\text{S1P}}_{3}^{-/-}$ *p* < 0.05, Figures 4A,C). S1P treatment effects were observed in cell capacitance, as it increased significantly in ${\text{S1P}}_{3}^{-/-}$ neurons, in the τ_AHP_, which decreased significantly in WT neurons, and in depolarization speed, which increased significantly in WT neurons (Table 4).

**Table 4:** **Basic electrophysiological properties of principal neurons in area CA3 in acute hippocampal slices from WT and ${\text{S1P}}_{3}^{-/-}$ mice before and after S1P application**.

**CA3**	**WT (*n* = 10)**		**${\text{S1P}}_{3}^{-/-}$ (*n* = 8)**		**Two-way repeated measures ANOVA**			**Bonferroni post-tests of S1P treatment effects**	
	**before S1P**	**after S1P**	**before S1P**	**after S1P**	**Interaction *p*-value**	**S1P treatment effect *p*-value**	**Genotype effect *p*-value**	**WT *p*-value**	**${\text{S1P}}_{3}^{-/-}$*p*-value**
Series resistance [MΩ]	22.20±0.8	27.28±3.8	23.19±0.9	24.18±1.8	0.3118	0.141	0.6956	>0.05	>0.05
Capacitance [pF]	120.3±5.5	131.0±7.5	126.1±9.1	149.4±13.3	0.0855	<0.0001^***^	0.3291	>0.05	<0.001^***^
Input resistance [MΩ]	354.1±33.6	347.9±40.4	257.0±37.7	275.1±39.7	0.5994	0.7966	0.1027	>0.05	>0.05
Resting membrane potential [mV]	−66.7±1.7	−70.6±1.4	−64.5±3.0	−67.1±2.3	0.2694	<0.0001^***^	0.2904	<0.001^***^	<0.05^*^
Rheobase [pA]	108.0±10.2	108.0±9.2	120.0±17.0	131.3±12.6	0.3724	0.3724	0.2886	>0.05	>0.05
AP no. in ramp based on 1x rheobase	12.5±2.4	7.9±2.0	7.3±1.7	7.3±1.3	0.0787	0.0787	0.2698	<0.05^*^	>0.05
Interspike intervals [ms]	169.6±29.8	278.9±37.2	365.2±161.1	200.3±28.3	0.0818	0.7101	0.4739	>0.05	>0.05
AP no. in ramp based on 2x rheobase	24.3±2.6	19.5±2.8	19.25±3.9	21.9±3.2	0.0156	0.4396	0.7522	<0.05^*^	>0.05
Interspike intervals [ms]	138.6±27.9	150.0±23.4	301.6±113.4	171.4±30.9	0.1125	0.1780	0.1883	>0.05	>0.05
AP threshold [mV]	−39.2±1.2	−39.1±1.0	−37.9±1.8	−37.5±2.1	0.4984	0.4350	0.5066	>0.05	>0.05
AP overshoot [mV]	59.7±0.4	60.5±0.7	59.1±1.5	59.6±1.6	0.6884	0.0695	0.6134	>0.05	>0.05
AP afterhyperpolarization [mV]	−52.0±1.0	−50.9±0.8	−50.0±1.1	−49.5±1.3	0.5119	0.0813	0.2339	>0.05	>0.05
τ_AHP_ [ms]	4.33±0.84	3.58±0.65	3.89±0.60	3.57±0.72	0.2621	0.0108^*^	0.8241	<0.05^*^	>0.05
AP amplitude [mV]	98.9±1.5	99.6±1.4	97.0±2.4	97.0±2.3	0.3140	0.2914	0.4055	>0.05	>0.05
AP duration [ms]	1.37±0.068	1.38±0.078	1.33±0.085	1.31±0.080	0.3878	0.7086	0.6441	>0.05	>0.05
Depolarization speed [mV/ms]	376.7±24.9	414.7±28.6	396.9±44.0	406.3±37.7	0.0560	0.0036^**^	0.9013	<0.01^**^	>0.05
Repolarization speed [mV/ms]	−59.5±2.4	−58.6±2.8	−59.4±3.1	−59.6±2.9	0.3914	0.6010	0.9163	>0.05	>0.05

Data are presented as mean ± SEM (Two-way repeated measures ANOVA with Bonferroni post-tests:

* **p* < 0.05*,

** **p* < 0.01*,

*** **p* < 0.001)*.

Additionally, we tested whether S1P application has an influence on eEPSC amplitude, as reported previously (Kanno et al., 2010). S1P application increased eEPSC amplitudes and the effect was observed both in WT neurons and neurons lacking S1P_3_, with a slightly weaker effect in ${\text{S1P}}_{3}^{-/-}$ neurons (relative eEPSC amplitude with S1P: WT: 1.94 ± 0.34%, ${\text{S1P}}_{3}^{-/-}$: 1.82 ± 0.34%; two-way repeated measures ANOVA: treatment *p* = 0.0018, genotype: *p* = 0.9667, interaction: *p* = 0.9012, Bonferroni post-tests: WT *p* < 0.01, ${\text{S1P}}_{3}^{-/-}$ *p* < 0.05, Figures 4B,D). We further analyzed eEPSC kinetics, comparing 10–90% rise and decay times. No significant differences in rise time were detected (WT control: 4.192 ± 0.557 ms, WT with S1P: 4.277 ± 0.583 ms, ${\text{S1P}}_{3}^{-/-}$ control: 3.66 ± 0.409 ms, ${\text{S1P}}_{3}^{-/-}$ with S1P: 3.465 ± 0.314 ms; two-way repeated measures ANOVA: interaction: *p* = 0.4722, treatment: *p* = 0.3408, genotype: *p* = 0.6841, Bonferroni post-tests: n.s.), whereas a significant increase of decay times after S1P was observed (WT control: 21.42 ± 1.979 ms, WT with S1P: 24.08 ± 2.124 ms, ${\text{S1P}}_{3}^{-/-}$ control: 20.86 ± 2.513 ms, ${\text{S1P}}_{3}^{-/-}$ with S1P: 21.46 ± 2.05 ms; two-way repeated measures ANOVA: interaction: *p* = 0.0716, treatment: *p* = 0.0109, genotype: *p* = 0.6147, Bonferroni post-tests: WT: *p* < 0.05, ${\text{S1P}}_{3}^{-/-}$: n.s.).

These results suggest that S1P_3_ receptor may contribute to modulation of neuronal excitability and synaptic transmission by S1P, but since no significant interaction between genotype and S1P treatment was detected, it is likely that the other S1P receptors contribute to the observed S1P treatment effects.

## Discussion

In this study, we provide evidence that the S1P_3_ receptor is associated with a distinct role in hippocampal function both *in vivo* and *in vitro*. Mice lacking S1P_3_ showed a significant impairment in an appetitively motivated spatial working memory task, but no impairments in other behavioral tests. The transgenic mouse model used in the present study suggests that S1P modulation of EPSCs does not require the presence of S1P_3_ receptors. However, principal neurons in hippocampal area CA3 lacking S1P_3_ exhibited significantly increased interspike intervals and significantly decreased input resistance, pinpointing to a critical role of the S1P_3_ receptor for the regulation of neuronal excitability in general.

Not only S1P_3_, but also S1P receptors 1, 2, 4, and 5 are expressed, with varying expression levels, in the different cell types of the central nervous system (for review see Choi and Chun, 2013) where they have been connected to various cellular functions including synaptic transmission (Soliven et al., 2011). In this study, we demonstrate that depletion of S1P_3_ does not lead to a significant change in mRNA expression levels of the other receptors. Since all other receptors are expressed, it is very likely that they are able to compensate for the loss of S1P_3_ as they can activate the same downstream signaling pathways.

Depletion of the S1P producing enzyme SphK1 with reduced S1P production induces an impairment of mossy fiber—CA3 LTP and a significant deficit in spatial reference memory (Kanno et al., 2010). In contrast, mice with a depletion of SphK2 display lower levels of hippocampal S1P, reduced histone acetylation and deficits in spatial memory as well as impaired contextual fear extinction (Hait et al., 2014). Thus, S1P and SphKs play specific roles in brain areas serving specific memory functions through intracellular S1P effects as well as signaling pathways downstream of S1P GPCRs.

Mechanisms of memory formation are most extensively studied in the hippocampus where SphK1 expression levels are high exclusively within hippocampal mossy fibers (Kanno et al., 2010) whereas *Sphk2* and *S1p*_2_ mRNA are expressed in the CA1–CA3 regions (Blondeau et al., 2007; Akahoshi et al., 2011; Kempf et al., 2014). Involvement of S1P_1_ in memory formation has not yet been demonstrated and mice with a global depletion of S1P_1_ are embryonically lethal (Liu et al., 2000). Since S1P_1_ expression is restricted to astrocytes and endothelial cells it is likely that this particular S1P receptor subtype affects synaptic plasticity since via indirect mechanism as astrocytes have been shown to regulate synaptic transmission and neuronal excitability (Volterra and Meldolesi, 2005; Brana et al., 2014).

${\text{S1P}}_{3}^{-/-}$ mice show signatures of augmented anxiety in the open field test and display a spatial working memory deficit in the eight arm radial maze, but no change in LTP formation at Schaffer collateral—CA1 synapses (Akahoshi et al., 2011). Interestingly though, inhibition of S1P_2_ leads to an increase in Schaffer collateral—CA1 LTP and a similar effect is observed when the newly discovered endogenous S1P_2_ ligand Nogo-A is neutralized (Kempf et al., 2014). In addition, we now demonstrate S1P_3_ expression in hippocampal neurons and this has been associated with a working memory deficit which was restricted to the performance of ${\text{S1P}}_{3}^{-/-}$ mice in an appetitively motivated T-maze task but none of the other tests performed. Mice displayed normal novelty and exploratory driven behavior and normal reference memory function. A possible explanation for the observed differences in behavior between the memory-related tests may be offered by a reduced appetitive motivation of ${\text{S1P}}_{3}^{-/-}$ mice. Another possibility could be a reduced capacity to learn a working memory task, which might also be due to changes in brain structures other than the hippocampus. Brain structures involved in spatial memory tasks include the temporal neocortex, basal forebrain, thalamus, prefrontal cortex, dorsal striatum, vestibular system, and cerebellum, and for example lesions of the entorhinal cortex lead to an impairment of learned alternation in rats (Lalonde, 2002).

Kanno et al were able to show that S1P_3_ translocates to presynaptic nerve terminals upon stimulation with S1P in acutely dissociated CA3 pyramidal neurons and leads to glutamate release at mossy fiber terminals (Kanno et al., 2010; Kanno and Nishizaki, 2011). Pharmacological evidence suggests that S1P and S1P_3_ are associated with LTP formation (Maggio et al., 2014). However, S1P significantly increased eEPSC amplitudes and eEPSC decay times both in WT and in ${\text{S1P}}_{3}^{-/-}$ mice, suggesting an increase in glutamate release, and possibly a reduction in non-NMDA receptor desensitization. Furthermore, S1P also decreased the resting membrane potential in both genotypes, leading to the conclusion that S1P_3_ shares redundant functions with other S1P receptors within CA3 neurons converging onto the same signaling pathways (Choi and Chun, 2013).

Although WT cells and cells lacking S1P_3_ were similar in their responses to S1P, significant differences in basic electrophysiological properties were detected. ${\text{S1P}}_{3}^{-/-}$ CA3 hippocampal principal neurons displayed significantly longer interspike intervals and decreased input resistances, indicating a general reduction of excitability, which might be the underlying cause for the reduced performance of ${\text{S1P}}_{3}^{-/-}$ mice in the working memory task of learned alternation in the T-maze.

We hypothesized that this reduced excitability might be caused by an involvement of potassium channels. So far no direct relationship between S1P signaling and potassium channels has been demonstrated in central neurons, but few publications from other areas of research link S1P receptors to several types of potassium channels. For example, S1P activates inwardly rectifying K^+^ currents (I_K.ACh_) in ventricular myocytes with the involvement of S1P_1_ and S1P_3_ (Landeen et al., 2008) and the SUR2/K_ir_6.2 channel via S1P_3_ in mouse ventricular fibroblasts (Benamer et al., 2009, 2011). Although a direct regulatory effect of S1P_3_ on potassium channel activity is not supported by the present results, this leaves three alternative possibilities as explanations for the significantly reduced input resistance and firing frequency in ${\text{S1P}}_{3}^{-/-}$ mice: first, other members of the large potassium channel family may be targeted by S1P_3_ receptor signaling similar to a study in human anaplastic thyroid cancer cells where S1P down-regulates Kv11.1 regulation via S1P_2_ (Asghar et al., 2012). Second, protein expression may be deregulated by mechanisms independent of transcription e.g., by microRNAs as shown for BK potassium channels (Pietrzykowski et al., 2008) or third, posttranslational modifications of ion channels such as protein phosphorylation or trafficking may be affected by S1P_3_ receptor depletion (Johnston et al., 2010).

## Conclusions

In the present study we provide evidence that the S1P_3_ receptor plays a specific role in the context of learning and memory and in a distinct subset of hippocampal principal neurons. S1P_3_ appears important for the formation of spatial working memory in the T-maze rewarded alternation paradigm through its influence on neuronal excitability.

## Authors contributions

Conceived and designed the experiments: DW, PM, SQ, LZ, IK, DB, ML, RH, CS, MK. Performed the experiments: DW, PM, SQ, LZ, IK, DB, CS, TK. Analyzed the experiments: DW, PM, SQ, LZ, DB, ML, RH, CS, MK, TK. Contributed reagents/materials/analytic tools: RP, RH, CS, MK. Wrote the manuscript: DW, PM, SQ, DB, RP, RH, CS, MK. All authors read and approved the final manuscript.

### Conflict of interest statement

The authors declare that the research was conducted in the absence of any commercial or financial relationships that could be construed as a potential conflict of interest.

## References

[B1] Akahoshi N. Ishizaki Y. Yasuda H. Murashima Y. L. Shinba T. Goto K.. (2011). Frequent spontaneous seizures followed by spatial working memory/anxiety deficits in mice lacking sphingosine 1-phosphate receptor 2. Epilepsy Behav. 22, 659–665. 10.1016/j.yebeh.2011.09.002 22019019

[B2] Antonucci F. Turola E. Riganti L. Caleo M. Gabrielli M. Perrotta C.. (2012). Microvesicles released from microglia stimulate synaptic activity via enhanced sphingolipid metabolism. EMBO J. 31, 1231–1240. 10.1038/emboj.2011.489 22246184 PMC3297996

[B3] Asghar M. Y. Viitanen T. Kemppainen K. Törnquist K. (2012). Sphingosine 1-phosphate and human ether-a'-go-go-related gene potassium channels modulate migration in human anaplastic thyroid cancer cells. Endocr. Relat. Cancer. 19, 667–680. 10.1530/ERC-12-0092 22889737

[B4] Asle-Rousta M. Kolahdooz Z. Oryan S. Ahmadiani A. Dargahi L. (2013). FTY720 (Fingolimod) Attenuates Beta-amyloid Peptide (Aβ42)-Induced impairment of spatial learning and memory in rats. J. Mol. Neurosci. 50, 524–532. 10.1007/s12031-013-9979-6 23435938

[B5] Benamer N. Fares N. Bois P. Faivre J.-F. (2011). Electrophysiological and functional effects of sphingosine-1-phosphate in mouse ventricular fibroblasts. Biochem. Biophys. Res. Commun. 408, 6–11. 10.1016/j.bbrc.2011.03.072 21420933

[B6] Benamer N. Maati H. M. O. Demolombe S. Cantereau A. Delwail A. Bois P.. (2009). Molecular and functional characterization of a new potassium conductance in mouse ventricular fibroblasts. J. Mol. Cell. Cardiol. 46, 508–517. 10.1016/j.yjmcc.2008.12.016 19166858

[B7] Bischofberger J. Engel D. Li L. Geiger J. R. Jonas P. (2006). Patch-clamp recording from mossy fiber terminals in hippocampal slices. Nat. Protoc. 1, 2075–2081. 10.1038/nprot.2006.312 17487197

[B8] Blondeau N. Lai Y. Tyndall S. Popolo M. Topalkara K. Pru J. K.. (2007). Distribution of sphingosine kinase activity and mRNA in rodent brain. J. Neurochem. 103, 509–517. 10.1111/j.1471-4159.2007.04755.x 17623044 PMC2639651

[B9] Brana C. Frossard M. J. Pescini Gobert R. Martinier N. Boschert U. Seabrook T. J. (2014). Immunohistochemical detection of sphingosine-1-phosphate receptor 1 and 5 in human multiple sclerosis lesions. Neuropathol. Appl. Neurobiol. 40, 564–578. 10.1111/nan.12048 23551178

[B10] Camprubí-Robles M. Mair N. Andratsch M. Benetti C. Beroukas D. Rukwied R.. (2013). Sphingosine-1-phosphate-induced nociceptor excitation and ongoing pain behavior in mice and humans is largely mediated by S1P3 receptor. J. Neurosci. 33, 2582–2592. 10.1523/JNEUROSCI.4479-12.2013 23392686 PMC6619173

[B11] Choi J. W. Chun J. (2013). Lysophospholipids and their receptors in the central nervous system. Biochim. Biophys. Acta 1831, 20–32. 10.1016/j.bbalip.2012.07.015 22884303 PMC3693945

[B12] Couttas T. A. Kain N. Daniels B. Lim X. Y. Shepherd C. Kril J.. (2014). Loss of the neuroprotective factor Sphingosine 1-phosphate early in Alzheimer's disease pathogenesis. Acta Neuropathol Commun 2:9. 10.1186/2051-5960-2-9 24456642 PMC3906863

[B13] Deacon R. M. J. Bannerman D. M. Kirby B. P. Croucher A. Rawlins J. N. P. (2002). Effects of cytotoxic hippocampal lesions in mice on a cognitive test battery. Behav. Brain Res. 133, 57–68. 10.1016/s0166-4328(01)00451-x 12048174

[B14] Deacon R. M. J. Rawlins J. N. P. (2006). T-maze alternation in the rodent. Nat. Protoc. 1, 7–12. 10.1038/nprot.2006.2 17406205

[B15] Hait N. C. Wise L. E. Allegood J. C. O'Brien M. Avni D. Reeves T. M.. (2014). Active, phosphorylated fingolimod inhibits histone deacetylases and facilitates fear extinction memory. Nat. Neurosci. 17, 971–980. 10.1038/nn.3728 24859201 PMC4256678

[B16] Ishii I. (2001). Selective loss of Sphingosine 1-Phosphate signaling with no obvious phenotypic abnormality in mice lacking its G Protein-coupled Receptor, LPB3/EDG-3. J. Biol. Chem. 276, 33697–33704. 10.1074/jbc.M104441200 11443127

[B17] Johnston J. Forsythe I. D. Kopp-Scheinpflug C. (2010). Going native: voltage-gated potassium channels controlling neuronal excitability. J. Physiol. (Lond.) 588, 3187–3200. 10.1113/jphysiol.2010.191973 20519310 PMC2976014

[B18] Kajimoto T. Okada T. Yu H. Goparaju S. K. Jahangeer S. Nakamura S.-I. (2007). Involvement of sphingosine-1-phosphate in glutamate secretion in hippocampal neurons. Mol. Cell. Biol. 27, 3429–3440. 10.1128/MCB.01465-06 17325039 PMC1899953

[B19] Kanno T. Nishizaki T. (2011). Endogenous sphingosine 1-phosphate regulates spontaneous glutamate release from mossy fiber terminals via S1P(3) receptors. Life Sci. 89, 137–140. 10.1016/j.lfs.2011.05.021 21683714

[B20] Kanno T. Nishizaki T. Proia R. L. Kajimoto T. Jahangeer S. Okada T.. (2010). Regulation of synaptic strength by sphingosine 1-phosphate in the hippocampus. Neuroscience 171, 973–980. 10.1016/j.neuroscience.2010.10.021 20950672

[B21] Kays J. S. Li C. Nicol G. D. (2012). Expression of sphingosine 1-phosphate receptors in the rat dorsal root ganglia and defined single isolated sensory neurons. Physiol. Genomics 44, 889–901. 10.1152/physiolgenomics.00053.2012 22805346 PMC3472456

[B22] Kempf A. Tews B. Arzt M. E. Weinmann O. Obermair F. J. Pernet V.. (2014). The sphingolipid receptor S1PR2 is a receptor for Nogo-a repressing synaptic plasticity. PLoS Biol. 12:e1001763. 10.1371/journal.pbio.1001763 24453941 PMC3891622

[B23] Kono M. Mi Y. Liu Y. Sasaki T. Allende M. L. Wu Y.-P.. (2004). The Sphingosine-1-phosphate Receptors S1P1, S1P2, and S1P3 function coordinately during embryonic angiogenesis. J. Biol. Chem. 279, 29367–29373. 10.1074/jbc.M403937200 15138255

[B24] Lalonde R. (2002). The neurobiological basis of spontaneous alternation. Neurosci. Biobehav. Rev. 26, 91–104. 10.1016/s0149-7634(01)00041-0 11835987

[B25] Landeen L. K. Dederko D. A. Kondo C. S. Hu B. S. Aroonsakool N. Haga J. H.. (2008). Mechanisms of the negative inotropic effects of sphingosine-1-phosphate on adult mouse ventricular myocytes. AJP: Heart Circ. Physiol. 294, H736–H749. 10.1152/ajpheart.00316.2007 18024550

[B26] Langeslag M. Malsch P. Welling A. Kress M. (2014). Reduced excitability of gp130-deficient nociceptors is associated with increased voltage-gated potassium currents and Kcna4 channel upregulation. Pflugers Arch. 466, 2153–2165. 10.1007/s00424-014-1443-0 24463703

[B27] Lein E. S. Hawrylycz M. J. Ao N. Ayres M. Bensinger A. Bernard A.. (2006). Genome-wide atlas of gene expression in the adult mouse brain. Nature 445, 168–176. 10.1038/nature05453 17151600

[B28] Liu Y. Wada R. Yamashita T. Mi Y. Deng C. X. Hobson J. P.. (2000). Edg-1, the G protein-coupled receptor for sphingosine-1-phosphate, is essential for vascular maturation. J. Clin. Invest. 106, 951–961. 10.1172/JCI10905 11032855 PMC314347

[B29] Maceyka M. Harikumar K. B. Milstien S. Spiegel S. (2012). Sphingosine-1-phosphate signaling and its role in disease. Trends Cell Biol. 22, 50–60. 10.1016/j.tcb.2011.09.003 22001186 PMC3253987

[B30] Maggio N. Itsekson Z. Ikenberg B. Strehl A. Vlachos A. Blatt I.. (2014). The anticoagulant activated protein C (aPC) promotes metaplasticity in the hippocampus through an EPCR-PAR1-S1P1 receptors dependent mechanism. Hippocampus 24, 1030–1038. 10.1002/hipo.22288 24753100

[B31] Mair N. Benetti C. Andratsch M. Leitner M. G. Constantin C. E. Camprubí-Robles M.. (2011). Genetic evidence for involvement of neuronally expressed S1P1 receptor in nociceptor sensitization and inflammatory pain. PLoS ONE 6:e17268. 10.1371/journal.pone.0017268.t003 21359147 PMC3040773

[B32] Obernosterer G. Martinez J. Alenius M. (2007). Locked nucleic acid-based *in situ* detection of microRNAs in mouse tissue sections. Nat. Protoc. 2, 1508–1514. 10.1038/nprot.2007.153 17571058

[B33] Okada T. Kajimoto T. Jahangeer S. Nakamura S.-I. (2009). Sphingosine kinase/sphingosine 1-phosphate signalling in central nervous system. Cell. Signal. 21, 7–13. 10.1016/j.cellsig.2008.07.011 18694820

[B34] Pietrzykowski A. Z. Friesen R. M. Martin G. E. Puig S. I. Nowak C. L. Wynne P. M.. (2008). Posttranscriptional regulation of BK channel splice variant stability by miR-9 underlies neuroadaptation to alcohol. Neuron 59, 274–287. 10.1016/j.neuron.2008.05.032 18667155 PMC2714263

[B35] Riganti L. Antonucci F. Gabrielli M. Prada I. Giussani P. Viani P.. (2016). Sphingosine-1-Phosphate (S1P) impacts presynaptic functions by regulating Synapsin I localization in the presynaptic compartment. J. Neurosci. 36, 4624–4634. 10.1523/JNEUROSCI.3588-15.2016 27098703 PMC6601834

[B36] Ruijter J. M. Ramakers C. Hoogaars W. M. H. Karlen Y. Bakker O. van den Hoff M. J. B.. (2009). Amplification efficiency: linking baseline and bias in the analysis of quantitative PCR data. Nucleic Acids Res. 37, e45–e45. 10.1093/nar/gkp045 19237396 PMC2665230

[B37] Soliven B. Miron V. Chun J. (2011). The neurobiology of sphingosine 1-phosphate signaling and sphingosine 1-phosphate receptor modulators. Neurology 76, S9–S14. 10.1212/WNL.0b013e31820d9507 21339490

[B38] Sørensen A. T. Rogelius N. Lundberg C. Kokaia M. (2011). Activity-dependent long-term plasticity of afferent synapses on grafted stem/progenitor cell-derived neurons. Exp. Neurol. 229, 274–281. 10.1016/j.expneurol.2011.02.008 21324317

[B39] Spijker S. (2011). Dissection of rodent brain regions, in Neuroproteomics Neuromethods, ed Li K. W. (Totowa, NJ: Humana Press), 13–26.

[B40] Strub G. M. Maceyka M. Hait N. C. Milstien S. Spiegel S. (2010). Extracellular and intracellular actions of sphingosine-1-phosphate. Adv. Exp. Med. Biol. 688, 141–155. 10.1007/978-1-4419-6741-1_10 20919652 PMC2951632

[B41] Takabe K. Paugh S. W. Milstien S. Spiegel S. (2008). “Inside-out” signaling of sphingosine-1-phosphate: therapeutic targets. Pharmacol. Rev. 60, 181–195. 10.1124/pr.107.07113 18552276 PMC2695666

[B42] Tuomi J. M. Voorbraak F. Jones D. L. Ruijter J. M. (2010). Bias in the Cq value observed with hydrolysis probe based quantitative PCR can be corrected with the estimated PCR efficiency value. Methods 50, 313–322. 10.1016/j.ymeth.2010.02.003 20138998

[B43] van Echten-Deckert G. Hagen-Euteneuer N. Karaca I. Walter J. (2014). Sphingosine-1-Phosphate: boon and bane for the brain. Cell. Physiol. Biochem. 34, 148–157. 10.1159/000362991 24977488

[B44] Volterra A. Meldolesi J. (2005). Astrocytes, from brain glue to communication elements: the revolution continues. Nat. Rev. Neurosci. 6, 626–640. 10.1038/nrn1722 16025096

[B45] Wittmann W. Schunk E. Rosskothen I. Gaburro S. Singewald N. Herzog H.. (2008). Prodynorphin-Derived Peptides Are Critical Modulators of Anxiety and Regulate Neurochemistry and Corticosterone. Neuropsychopharmacology 34, 775–785. 10.1038/npp.2008.142 18800067 PMC2873573

[B46] Yamaguchi F. Tokuda M. Hatase O. Brenner S. (1996). Molecular cloning of the novel human G protein-coupled receptor (GPCR) gene mapped on chromosome 9. Biochem. Biophys. Res. Commun. 227, 608–614. 10.1006/bbrc.1996.1553 8878560

[B47] Zhang G. Contos J. J. Weiner J. A. Fukushima N. Chun J. (1999). Comparative analysis of three murine G-protein coupled receptors activated by sphingosine-1-phosphate. Gene 227, 89–99. 10.1016/s0378-1119(98)00589-7 9931453

[B48] Zhang Y. H. Fehrenbacher J. C. Vasko M. R. Nicol G. D. (2006). Sphingosine-1-phosphate via activation of a G-protein-coupled receptor(s) enhances the excitability of rat sensory neurons. J. Neurophysiol. 96, 1042–1052. 10.1152/jn.00120.2006 16723416

