# Supplementary material for: Ablation of Sphingosine 1-Phosphate Receptor Subtype 3 Impairs Hippocampal Neuron Excitability In vitro and Spatial Working Memory In vivo
Source: Front Cell Neurosci. 2016 Nov 7;10:258. doi: 10.3389/fncel.2016.00258 (PMC5097928; doi:10.3389/fncel.2016.00258)
Supplement: Supplementary file 1 [file DataSheet1.DOCX]

**Supplementary Table 1 Mean PCR efficiencies of primers**

| **Gene** | **Assay identifier** | **Target/Hkg** | **Mean PCR efficiency** |
| --- | --- | --- | --- |
| *Hprt* | Mm00446968_m1 | Hkg | 1.816 |
| *Kcna4* | Mm01336166_m1 | Target | 1.854 |
| *Kcnc3* | Mm00434614_m1 | Target | 1.777 |
| *Kcnc4* | Mm00521443_m1 | Target | 1.947 |
| *Kcnd2* | Mm01161732_m1 | Target | 1.835 |
| *Kcnd3* | Mm01302126_m1 | Target | 1.812 |
| *Kcnj3* | Mm00434618_m1 | Target | 1.934 |
| *Kcnj6* | Mm01215650_m1 | Target | 1.807 |
| *Kcnj8* | Mm00434620_m1 | Target | 1.954 |
| *Kcnj9* | Mm00434622_m1 | Target | 1.798 |
| *S1pr1* | Mm02619656_s1 | Target | 1.696 |
| *S1pr2* | Probe Assay: S1pr2, Mouse | Target | 1.807 |
| *S1pr3* | Mm04229896_m1 | Target | Not calculated |
| *S1pr4* | Mm00468695_s1 | Target | 1.935 |
| *S1pr5* | Mm02620565_s1 | Target | 1.827 |
| *Sdha* | Mm01352363_m1 | Hkg | 1.752 |
| *Tfrc* | Mm00441941_m1 | Hkg | 1.835 |

Hkg: housekeeping gene
